# Complete genome sequence of *Acinetobacter guillouiae* CE15 isolated from decaying bamboo culms

**DOI:** 10.1128/mra.00737-25

**Published:** 2025-10-03

**Authors:** Bei Huang, Yu Sun, Yu-Qin Luo, Xiao-Long He, Cheng-Jing Ren, Han-Jia Liu, Jiang-Long Guo, Shu-Yu Tan, Li-Hua Lei, Jin-Ping Deng, Shuai-Bo Han

**Affiliations:** 1 School of Chemical and Materials Engineering, National Engineering & Technology Research Center of Wood-Based Resources Comprehensive Utilization, Zhejiang A&F University, Hangzhou, China; 2 FAW-Volkswagen Automotive Co., Ltd. 668086 , Changchun, China; 3 Jiangshan Fenghe Home Furnishing Co., Ltd, Quzhou, China; 4 Zhejiang Kesida New Material Technology Co., Ltd, Lishui, China; 5 Zhejiang Provincial Key Laboratory of Germplasm Innovation and Utilization for Garden Plants, Key Laboratory of National Forestry and Grassland Administration on Germplasm Innovation and Utilization for Southern Garden Plants, School of Landscape and Architecture, Zhejiang A&F University 562606, Hangzhou, China; Portland State University, Portland, Oregon, USA

**Keywords:** *Acinetobacter*, genome sequencing, decaying bamboo

## Abstract

*Acinetobacter*, a genus of gram-negative bacteria, is predominantly found in aquatic and terrestrial environments. In this study, we present the complete genome sequence of *Acinetobacter guillouiae* CE15, which is characterized by a total length of 4,661,234 base pairs, GC content of 38.29%, and the presence of 4,220 protein-coding genes.

## ANNOUNCEMENT

In environments with abundant moisture and suitable temperatures, bamboo is vulnerable to mold and decay induced by various microbes (1, 2). Unlike fungi, bacteria were largely unknown during the biodeterioration of bamboo. The genus *Acinetobacter* is heterogeneous and highly diverse metabolically as well as physiologically (3). Certain *Acinetobacter* species exhibit remarkable adaptability to extreme environments, demonstrating versatile metabolic capacities, including the degradation of diverse aromatic compounds (4).

Strain CE15 was isolated in March 2023 from decaying bamboo samples collected from the campus of Zhejiang A&F University (119°44′01′′E, 30°15′29′′N). The isolation, purification, and molecular identification of strain CE15 were employed according to the previous study (5). Specifically, bamboo extract liquid was prepared by placing 50 g of fresh bamboo culms into 1 L of distilled water and boiling it for 10 min. The 16S rDNA sequence of strain CE15 (GenBank accession number PP702142) showed 99.18% similarity with *Acinetobacter guillouiae* CIP 63.46.

A single colony of CE15 was incubated and cultured overnight in LB broth (6) at 30°C. Genomic DNA was extracted utilizing the Bacterial Genomic DNA Rapid Extraction Kit (Solarbio, Beijing, China). The genome was sequenced employing a combination of the Oxford Nanopore PromethION platform for long-read sequencing and the Illumina Novaseq 6000 platform for paired-end sequencing by Wuhan Benagen Technology Co., Ltd. (Hubei, P.R. China). The preparation of the DNA library for Illumina and Oxford Nanopore platform sequencing was followed by the methods outlined by Li et al. (7). All reads were provided as FASTQ files [Illumina, 7,970,019×2 raw pair reads (2,391,005,700 bases, with raw Q30 of 92.311%); Oxford Nanopore Technologies (ONT), 104,084 reads and (989, 981, 977 bases, the largest, average length and reads N50 value of 168,755 bp, 9,511.4 bp, and 18,608 bp)]. The raw data generated by Illumina sequencing was clipped using fastp v0.23.0 (8) to obtain high-quality clean data.

Nanopore sequencing reads were extracted, subjected to base calling and demultiplexing, and subsequently trimmed using Porechop (version 0.2.4, https://github.com/rrwick/porechop) with a minimum quality score threshold of 5. The sequencing depths achieved for Illumina and ONT were 503.2 and 213.53, respectively. The base calling was performed utilizing Guppy version 5.0.16 (9) in high-accuracy mode. Genome assembly was executed using Unicycler version 0.5.0 (10), and the quality of the assembly was assessed with CheckM version 1.1.10 (11). Pilon software (12) was employed to align Illumina short reads for correction purposes. Genome annotation was carried out using the NCBI Prokaryotic Genome Annotation Pipeline (13) and the Rapid Annotation using Subsystem Technology server (14). All software tools were utilized with default parameters unless otherwise indicated.

The genome of strain CE15 was assembled as one chromosome without a plasmid, with a total length of 4,661,234 bp and contains 4,220 protein-coding genes, 81 tRNA genes, 21 rRNA genes, and an average GC content of 38.29%.

## Data Availability

The genome sequence of strain CE15 is available in GenBank under accession numbers CP192737. The raw sequences are available under Sequence Read Archive (SRA) accession numbers SRR33997040 (Illumina) and SRR33997039 (ONT).
